# Bovine Dermal Collagen Matrix Promotes Vascularized Tissue Generation Supporting Early Definitive Closure in Full-Thickness Wounds: A Pre-clinical Study

**DOI:** 10.7759/cureus.81517

**Published:** 2025-03-31

**Authors:** Katie A Bush, Barbara A Nsiah, Jayson W Jay, Rachel A Penny, Sohail Jahid, Ghaidaa Y Kashgari, Niraj K Doshi, Ian L Valerio

**Affiliations:** 1 Scientific and Medical Affairs, AVITA Medical, Valencia, USA; 2 Research and Development, AVITA Medical, Valencia, USA; 3 Plastic and Reconstructive Surgery, Massachusetts General Hospital, Boston, USA

**Keywords:** advanced wound care, burn wound care, dermal matrix, excision of burn and grafting, full thickness wounds, post-surgical wounds, regenerative material, split-thickness skin grafting, traumatic wounds, xenogeneic dermal matrix

## Abstract

Objective

Dermal matrices are commonly used to manage full-thickness wounds, impacting both functional and aesthetic outcomes. However, standard materials typically require 14-28 days to develop sufficient tissue for autografting. This study aimed to assess the potential of a novel bovine dermal collagen matrix (BDCM) to decrease the time required to develop tissue for autografting for accelerating the timeline for definitive wound closure.

Methods

Full-thickness excisional wounds were surgically created on Yorkshire pigs and treated with crosslinked collagen-based dermal matrices, including a bovine dermal collagen matrix (BDCM; Cohealyx, Collagen Matrix Inc., Oakland, NJ), a collagen-glycosaminoglycan matrix (ColGAG; Integra Bilayer Wound Matrix, Integra Life Sciences, Plainsboro, NJ), or a fish skin graft matrix (FSG; Kerecis Graft Guide, Isafjorour, Iceland). Seven days after application, matrix attachment and infection status were assessed, along with histological analyses of cellular infiltration, collagen deposition, and angiogenesis. The wound beds were then autografted and monitored for an additional 35 days to evaluate wound healing parameters, including skin graft take, re-epithelialization, and wound contraction.

Results

Seven days post-autografting, autograft take was 96.88 ± 7.04% for BDCM-treated wounds compared to 85.00 ± 34.10% and 68.00 ± 27.97% for ColGAG and FSG-treated wounds, respectively. The coefficient of variation was 7% for BDCM-treated wounds compared to approximately 40% for ColGAG and FSG-treated wounds. Histological analysis revealed that BDCM-treated wounds contained a persisting collagen scaffold that allowed cellular infiltration and angiogenesis with no infection. At 42 days, BDCM showed significantly less wound contraction (p<0.05) compared to the other dermal matrix-treated wounds.

Conclusion

Bovine dermal collagen matrix enables rapid cellular infiltration and vascularized tissue deposition, supporting highly consistent autograft take at seven days. The possibility of reducing time to definitive closure has the potential to significantly improve clinical outcomes of acute full-thickness wounds and lower the overall cost of treatment.

## Introduction

Dermal matrices are designed to support dermal-like tissue generation and improve autograft outcomes for acute full-thickness wounds. These matrices provide a three-dimensional scaffold to support cellular infiltration, proliferation, and repopulation with blood vessels, preparing the wound bed for autografting. They are effective in supporting autograft survivability over exposed or poorly vascularized tissues as well as addressing large tissue deficits resulting from traumatic injuries or infections. Additionally, they play a critical role in improving aesthetic and functional results in scar revision surgeries [1,2].

In clinical practice, dermal matrices are applied to the wound bed after surgical excision of the full-thickness injury. Once a vascularized tissue is formed in the wound bed, an autografting procedure is performed for definitive closure, typically occurring between 14 and 28 days [3-5]. While this timeline benefits some patients, prolonged waiting for matrix integration and blood vessel formation can negatively impact outcomes, particularly when skin is available for autografting or donor-sparing techniques, such as autologous skin cell suspension, can be employed. Delays in closure may increase infection risk and pain and result in poor scar outcomes [6-8].

The composition and microarchitecture of dermal matrices directly influence tissue generation and time to wound closure [9]. Available dermal matrices vary in composition, including decellularized xenogeneic or allogeneic tissues, as well as bioengineered materials from biological or synthetic sources [10]. After implantation, host cells, including fibroblasts and endothelial cells, infiltrate the matrix, initiating collagen deposition and angiogenesis [11]. This matrix integration is linked to pore size, with an optimal size required to facilitate cell migration and vascular tissue ingrowth [12].

To decrease the time between dermal matrix application and autografting, a matrix composed of native collagen types I and III from young bovine dermis was bioengineered with biological cues, defined microarchitecture, and controlled degradation profile. Collagen types I and III are integral to the wound healing process, supporting tissue integrity and function [13]. Type I collagen maintains structural integrity, while type III stimulates collagen fiber production and regulates growth [14-16]. The absence of type III collagen has been linked to scar formation [17]. The tailored porosity and degradation profile of the matrix was designed to support rapid tissue ingrowth and vascular deposition.

The purpose of this pre-clinical study was to evaluate the ability of the bovine dermal collagen matrix (BDCM) to generate vascularized tissue in a full-thickness wound and support autografting at seven days. Commercially available crosslinked collagen-based materials, one bioengineered and one decellularized from a xenogeneic source, were used for comparison. A porcine full-thickness excisional wound model was used, as it is the most clinically relevant model system for wound healing [18]. The wounds were assessed over six weeks for inflammation, infection, autograft take, re-epithelialization, revascularization, and contraction using visual observation, histopathology, and planimetry.

This article was previously presented as a meeting abstract at the Boswick Burn & Wound Care Symposium on January 26, 2025.

## Materials and methods

Study design

The animal protocol used for this study was conducted under the oversight of the USDA, Institutional Animal Care and Use Committee (IACUC), and the Association for Assessment and Accreditation of Laboratory Animal Care International (AALAC), ensuring adherence to the Animal Welfare Act, ethical guidelines, and best practices.

All procedures were carried out under proper anesthesia and pain relief. Yorkshire pigs weighing approximately 30 kg at Day 0 were utilized. Six 16 cm^2^ full-thickness defects were created on the dorsum of the animals and dermal matrices were applied, randomly distributed among pigs and wound sites. On Day 7, the wound bed was biopsied, debrided, and autografted. Animals were observed for up to 35 days post-autografting, with biopsies taken on the day of sacrifice. Throughout the study, dressing changes occurred every 3±1 days until Day 28. After Day 28, weekly dressing changes were implemented.

Wound creation

Three days before wound creation, 16 cm^2^ wound outlines were tattooed on the dorsum of the animal with a 4 cm distance between each wound. On Day 0, prior to excision, pigs were shaved, washed, and prepped with betadine. Full-thickness wounds were created by surgically excising the entire epidermal, dermal, and adipose layers. The thickness of the wounds was approximately 8 mm. In total, 23 wounds were treated in this study.

Dermal matrix application

Three different types of dermal matrices were used: Cohealyx™ (Collagen Matrix Inc., Oakland, NJ, bovine dermal collagen matrix, BDCM), Integra® Bilayer Matrix (Integra Life Sciences; Plainsboro, NJ, collagen-glycosaminoglycan matrix, ColGAG), and Kerecis® Graft Guide (Kerecis; Isafjorour, Iceland; Fish Skin Graft, FSG). Each product was hydrated in saline and placed in the wound bed. The bovine dermal collagen matrix is a 3 mm thick single layer composed of purified bovine dermal collagen type I and type III. ColGAG is a 1.2 mm thick bilayer matrix containing a thin top layer composed of silicone and a primary wound contacting layer composed of bovine tendon collagen I and shark cartilage glycosaminoglycans. FSG is a 0.61 mm thick single layer derived from decellularized piscine skin containing collagens, elastins, glycosaminoglycans (GAGs), and omega 2 fatty acids. The sample size included BDCM n=8, ColGAG n=10, and FSG n=5 for visual observations and wound tracings. For histological characterization, pre-wound bed preparation biopsies were taken with n=5 for each treatment group.

Vicryl 3-0 sutures were used to secure dermal matrices into the wound bed (Ethicon US, LLC; Raritan, NJ). Following dermal matrix application, all wounds received the following dressings: Telfa™ Clear (Cardinal Health™; Dublin, OH), Xeroform® Petrolatum Dressing (Dukal Corporation; Ronkonkoma, NY), Curity™ gauze (Covidien/Medtronic; Minneapolis, MN), and a tie-over gauze bolster. Protective and outer dressings included Mextra Superabsorbant (Molnlycke Health Care US LLC; Norcross, GA), Ioban™ 2 Sterile Antimicrobial Incise Surgical (3M™; Saint Paul, MN), and Elastikon Elastic Tape (BSN Medical Inc; Charlotte, NC). 

Matrix Integration

Post-dermal matrix application, matrix integration was scored through a visual assessment of the percentage of the matrix attached to the wound bed and containing tissue within the matrix. Dermal matrix integration was scored based on the following scale: 0: 0%, 1: 1-25%, 2: 26-50%, 3: 51-74%, 4: 75%-95%, or 5:≥95% matrix filled with tissue, red material observed. 

Infection

Gross wound infection was assessed visually. Wounds that had areas of purulent drainage or discharge were scored as infected on the following scale: 0: no infection, 1: minimal infection (1-25% of the wound), 2: mild infection (26-50% of the wound), 3: moderate infection (51-74% of the wound), or 4: marked or severe infection (≥75% of the wound). 

Autografting

Seven days post-dermal matrix application, a meshed split-thickness autograft and autologous skin cell suspension (ASCS) were prepared and applied to each wound. Skin was collected from the flank of the pig using a dermatome (Zimmer Biomet; Warsaw, IN). Prior to skin harvest, the area of skin collection was shaved and washed with soap and water. Skin was harvested at a depth of 0.010-0.012” for autografts and at a depth of 0.006-0.008” for ASCS. Following collection, skin used for autografts was meshed at a ratio of 3:1 using a mesher (Zimmer Biomet). Autologous skin cell suspension was prepared following manufacturer’s guidelines (RECELL® Autologous Cell Harvesting Device, AVITA Medical; Valencia, CA). The meshed autografts were applied to achieve comparable expansion across groups, within the limits of surgical handling. All grafts were secured in place using sutures to maintain consistent contact with the wound surface. Autologous skin cell suspension was applied by dripping the suspension over the autograft. The same bandaging method previously described was used.

The autograft ratio (autograft area relative to total wound area, or dosing) was measured in Photoshop® (Adobe Systems, San Jose, CA) by precisely tracing the graft with a pseudo-color on a separate layer using an Intuos Pro creative pen tablet (Wacom, Vancouver, WA) and measuring the corresponding pixels. The same procedure was applied to the total wound area by tracing the outline of the wound bed following the tattoo. The percentage of autografted area to total wound area was then calculated for each wound to determine dosing.

Split-thickness autograft take

Seven days post-autografting, autograft take was visually assessed by determining the percentage of the autograft attached to the wound bed.

Re-epithelialization

Re-epithelialization was visually assessed by examining the presence of epithelium throughout the full wound area over the course of the study.

Wound size and contraction

Wound size was collected by tracing the tattoo outline of each wound. From the wound tracings, the area was determined by digital planimetry using ImageJ (National Institute of Health; Bethesda, MA). Wound contraction was calculated by subtracting the measured wound area (Day X) from the original wound area (Day 0) and then normalizing to the original wound area (Day 0). To obtain the percentage, this value was multiplied by 100.

Histopathological evaluation

The wound bed was biopsied at 7 and 42 days post-dermal matrix application. Tissue samples were processed, embedded in paraffin, and sectioned at 5 µm for histopathological evaluation. Tissue sections were mounted on slides and stained with hematoxylin and eosin (H&E) (Surgipath® Harris Hematoxylin Nuclear Stains, Surgipath® Eosin Secondary-Counter Stain, Leica Biosystems; Nussloch, Germany). Stained tissue sections were digitally scanned and blindly assessed by an independent board-certified veterinary pathologist. 

Quantification of cell infiltration was performed by an analysis of hematoxylin using image H&E-stained sections in each treatment group. To standardize evaluation, a predefined region of interest for each tissue section was analyzed using ImageJ with the Color Deconvolution2 plug-in (NIH, https://imagej.nih.gov/ij/download, https://imagej.net/plugins/colour-deconvolution). Tissue sections were also stained using Trichrome One-Step Stain Kit Procedure (StatLab, American MasterTech Scientific; McKinney, TX) and Pico-Sirius Red Stain Pint Kit (StatLab) for collagen evaluation. Using an Aperio AT2, whole slide scanning was performed. Images of Picosirius Red-stained tissue sections were utilized to quantify collagen presence in each treatment group. To standardize evaluation, a predefined region of interest for each tissue section was analyzed using ImageJ.

Immunohistochemical evaluation

Immunohistochemistry was performed using a Bond Rx Auto stainer (Leica Biosystems; Nussloch, Germany). For identification of blood vessels, DAB rabbit polyclonal CD31 primary antibody (1:50) (28364, Abcam; Cambridge, UK) and BOND Polymer Refine Detection were used according to the manufacturer’s recommendations (Leica). Using an Aperio AT2, whole slide scanning was performed. Images of CD31-stained tissue sections were utilized to quantify blood vessels in each treatment group. To standardize evaluation, a predefined region of interest for each tissue section was analyzed using ImageJ. For the identification of immune cells, DAB rabbit polyclonal CD45 primary antibody (1:100) (10558, Abcam) and BOND Polymer Refine Detection were used according to manufacturer’s recommendations (Leica). Using an Aperio AT2, whole slide scanning was performed. Images of CD45-stained tissue sections were utilized to quantify immune cells in each treatment group and analyzed using ImageJ.

Statistical analysis

Results are reported as percentages and mean ± standard deviation. GraphPad Prism software (GraphPad Software; Boston, MA) was used to perform analysis of variance (ANOVA) tests to determine differences between treatment groups. A multiple-comparisons test was conducted using Tukey’s multiple-comparisons testing. Tests with p-values less than 0.05 were deemed statistically significant.

## Results

Dermal matrix integration

**Figure 1:**
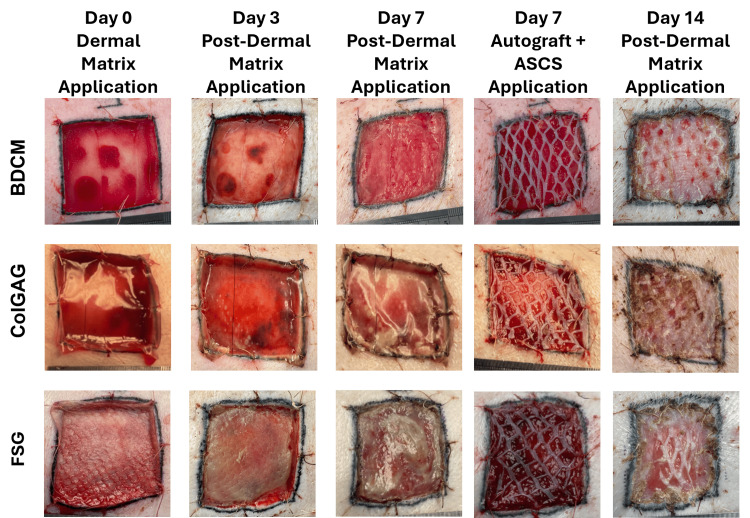
Visual assessment of wound bed preparation and autograft take using three collagen-based dermal matrices Representative images of bovine dermal collagen matrix (BDCM), collagen-glycosaminoglycan matrix (ColGAG), and fish skin graft (FSG)-treated full-thickness wounds. Dressing changes occurred on Day 3. All wounds were grafted at 7 days using a 3:1 meshed autograft with the application of autologous skin cell suspension (ASCS). Variations in wound bed preparation and autograft take were visually observed.

Upon application to the excised full-thickness wound bed, BDCM and ColGAG quickly absorbed the blood and exudate present in the wound. The FSG absorbed some of the fluid, but noticeably less than the BDCM and ColGAG materials (Figure 1). The BDCM easily conformed to the wound bed, whereas ColGAG and FSG required manipulation and trimming to achieve complete contact with the underlying surface (Figure 1).

Integration of the dermal matrices differed over the first seven days (Figure 1). Visual observation of the BDCM-treated wounds revealed a robustly vascularized tissue bed, where the dermal matrix could be seen with red tissue throughout. The ColGAG-treated wounds had areas of red and yellow color and no integration in areas with purulent drainage. Wounds treated with FSG had a caramelized appearance with areas of the matrix visually not integrated and areas sloughing off.

**Figure 2:**
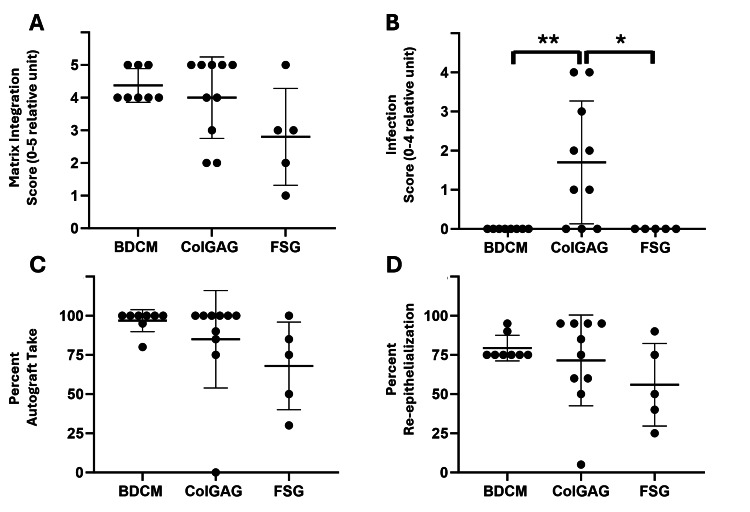
Matrix Integration, infection, percent autograft take, and percent re-epithelialization of three collagen-based dermal matrices Matrix integration varied for each dermal matrix with 100% bovine dermal collagen matrix (BDCM), 80% collagen-glycosaminoglycan matrix (ColGAG), and 20% fish skin graft (FSG)-treated wounds achieving ≥75% matrix integration (≥4) (A). Infection was seen in 70% of ColGAG wounds, whereas no infection was detected in BDCM or FSG wounds. * indicates p=0.0206 and ** indicates p=0.0075 (B). Autograft take was found to be 96.88 ± 7.04%, 85.00 ± 34.10%, and 68.00 ± 27.97% for BDCM, ColGAG, and FSG-treated wounds, respectively (C). At seven days post-autografting, the percentage of wounds achieving ≥75% re-epithelialization was found to be 100%, 60%, and 40% for BDCM, ColGAG, and FSG-treated wounds, respectively. Mean re-epithelialization was 79.38 ± 8.21%, 71.50 ± 28.97%, and 56.00 ± 23.54% for BDCM, ColGAG, and FSG-treated wounds, respectively (D). Data are reported as individual points and mean ± standard deviation.

Matrix integration was assessed, and 100% of BDCM-treated wounds, 80% of ColGAG-treated wounds, and 20% of FSG-treated wounds achieved ≥75% matrix integration (Scored as 4 and above) (Figure 2A). No infection was observed in the BDCM- or FSG-treated wounds; however, it was seen in 70% of the ColGAG-treated wounds (Figure 2B). The relative amount of infection was found to significantly differ between the BDCM and ColGAG, and FSG and ColGAG matrices (BDCM p=0.0075 and FSG p=0.0206).

Wound beds were prepared for autografting by debriding any non-integrated dermal matrix. BDCM-treated wounds required minimal debridement. ColGAG and FSG required more debridement to remove areas of non-integrated matrix and infection (ColGAG).

Autograft dosing and take

Autografts were meshed at a ratio of 3:1 and randomly applied to the prepared wound beds. The percentage of wound area covered by an autograft (dosing) was 48.8 ± 7.8%, 50.0 ± 5.5%, and 51.1% ± 6.1% for BDCM, ColGAG, and FSG treated wounds, respectively. No significant differences were observed (p=0.842). 

Seven days post-autografting, autograft take was found to vary based on the dermal matrix used. On average, 96.88 ± 7.04%, 85.00 ± 34.10%, and 68.00 ± 27.97% autograft take was reported for BDCM, ColGAG, and FSG, respectively (Figure 2C). Autograft take was most consistent in wounds treated with BDCM, with a coefficient of variance of 7%, while FSG and ColGAG coefficient of variances were approximately 40%. Moreover, 87.5% of BDCM wounds had autograft take ≥95%, whereas 60% of ColGAG wounds and 20% of FSG wounds had autograft take ≥95%. No significant differences were observed, likely due to the variability in the ColGAG and FSG treatment groups.

Re-epithelialization

Seven days post-autografting, 100% of the BDCM-treated wounds achieved ≥75% re-epithelialization, and 60% and 40% of the ColGAG and FSG-treated wounds achieved this level, respectively (Figure 2D). BDCM, ColGAG, and FSG averaged 79.38 ± 8.21%, 71.50 ± 28.97%, and 56.00 ± 23.54% re-epithelialization, respectively. In areas with autograft take, the autografts remained healthy and matured throughout the wound healing process in areas in which the matrix was integrated at the time of application. At 21 days post-autograft, 100% of BDCM-treated wounds had wound closure (>95% re-epithelialization), and 90% and 80% of ColGAG- and FSG-treated wounds achieved wound closure, respectively. At 42 days, all wounds were closed.

Histological analysis

**Figure 3:**
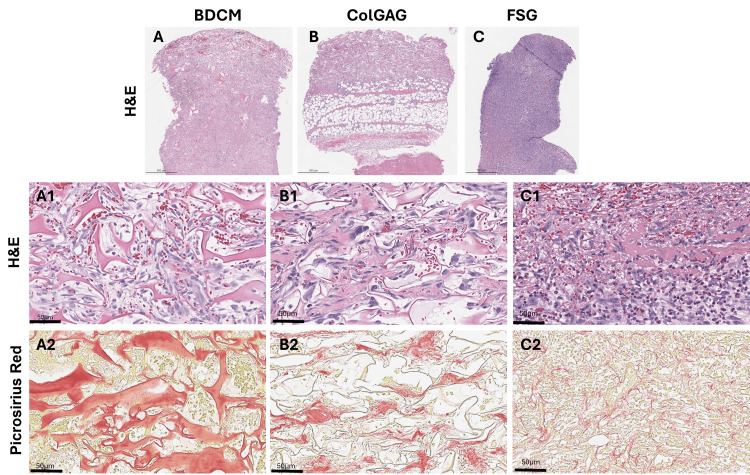
Histological evaluation of cellular infiltration and matrix structure seven days post-dermal matrix application Hematoxylin & eosin (H&E) was used to evaluate cellular infiltration and matrix structure seven days post-matrix implantation. Representative images of H&E-stained tissue sections show integrated dermal matrix fragments (eosin-stained pink) within the wound bed for bovine dermal collagen matrix (BDCM) (A), collagen-glycosaminoglycan matrix (ColGAG) (B), and fish skin graft (FSG)-treated wounds (C) (scale bar = 500 µm). Higher-magnification images taken from the top of the wound bed highlight dermal matrix regions with cellular infiltration (hematoxylin-stained purple) (A1–C1) (scale bar = 50 µm). Picrosirius red staining was used to detect the presence of collagen in tissue sections seven days post-matrix implantation. Higher-magnification images of the upper wound bed (A2–C2) highlight collagen-rich areas (red staining) for each dermal matrix (scale bar = 50 µm).

Seven days following dermal matrix application, the dermal matrices were visible in histological sections of the wound beds detected through the use of H&E stain (eosinophilic pink stain indicates a protein-rich structure) (Figures 3A-3C, 3 panels A1-C1). Cells appeared closely associated with the matrices. Picrosirius red staining specifically indicates the presence of collagen in each wound (Figures 3, panels A2-C2).

**Figure 4:**
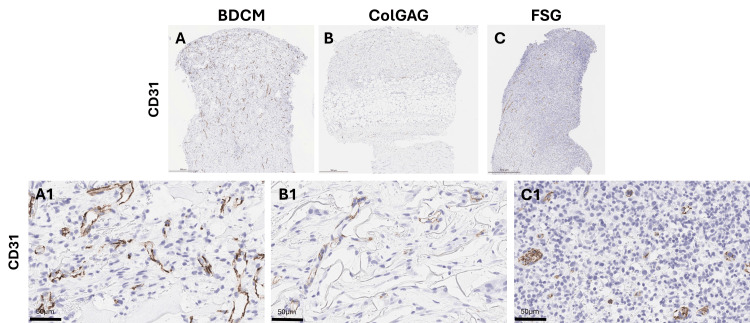
Histological evaluation of revascularization seven days post-dermal matrix application Immunohistochemistry was performed to visualize blood vessels within the wound bed using CD31 (brown stain). Representative images of CD31+ staining in biopsied tissue sections seven days after dermal matrix application are shown for bovine dermal collagen matrix (BDCM) (A), collagen-glycosaminoglycan matrix (ColGAG) (B), and fish skin graft (FSG)-treated wounds (C) (scale bar = 500 µm). Higher-magnification representative images (A1–C1) highlight blood vessels within each wound bed (scale bar = 50 µm).

Angiogenesis was evaluated through immunohistochemistry utilizing CD31 (Figure 4).

**Figure 5:**
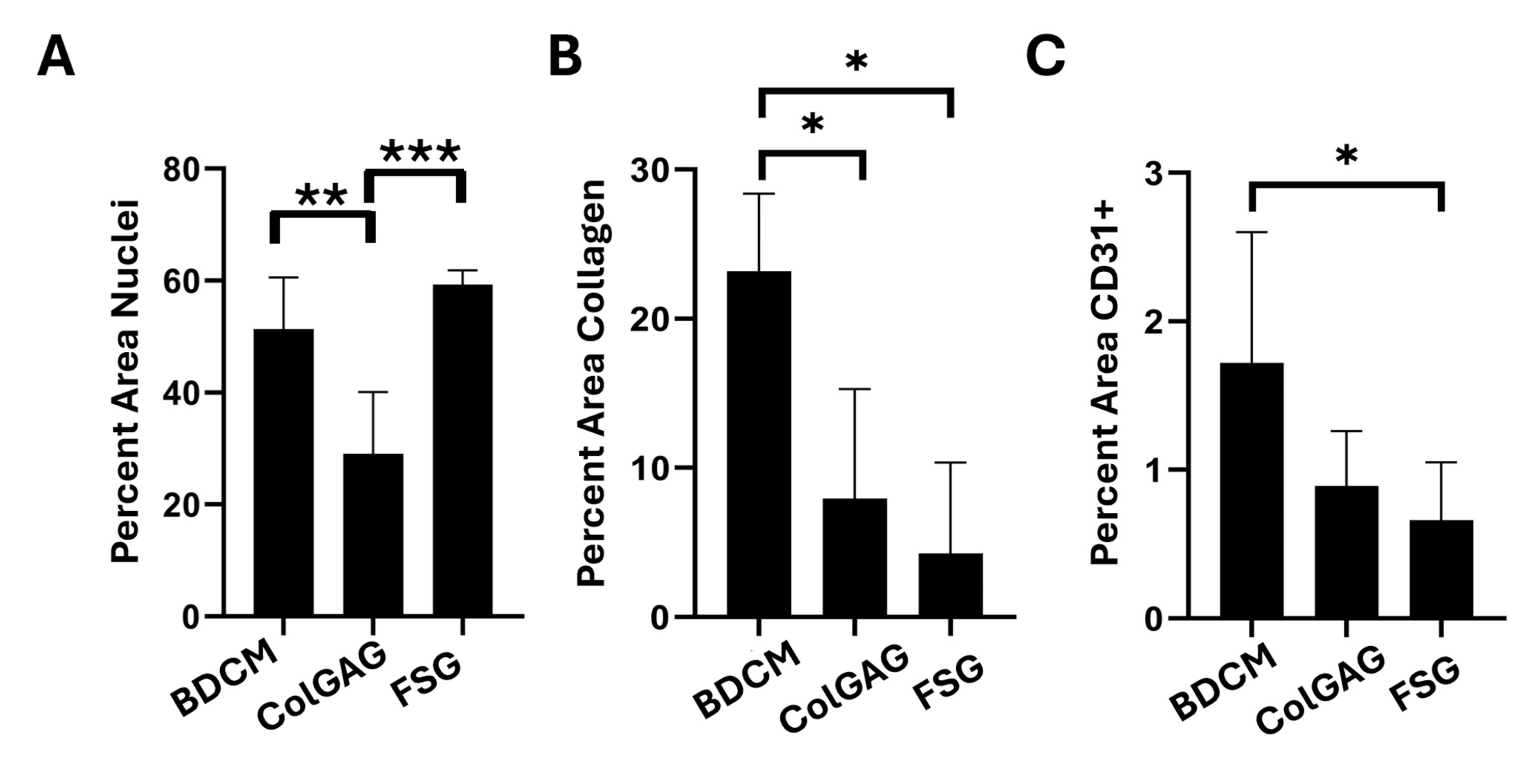
Quantification of cell infiltrate and collagen seven days post-dermal matrix application Quantification of cells within the wound bed using H&E staining indicates a significant increase of cellular density within the fish skin graft (FSG) (59.27 ± 2.54%) and bovine dermal collagen matrix (BDCM) (51.36 ± 9.21%)-treated wounds compared to the collagen-glycosaminoglycan matrix (ColGAG) (29.02 ± 11.05%)-treated wounds. ** indicate p<0.005 and *** indicates p<0.0005 (A). Picrosirius red staining was used to detect the percent area collagen. A significant difference was detected between the percent area collagen in the wound bed between the BDCM and FSG-treated wounds and the BDCM and ColGAG-treated wounds. The percent area collagen was 23.18 ± 5.21%, 7.94 ± 7.34%, and 4.27 ± 6.10% for BDCM, ColGAG, and FSG-treated wounds, respectively. * indicates p<0.05 (B). Angiogenesis was evaluated through staining of histological sections with CD31. Bovine dermal collagen matrix, ColGAG, and FSG percent area CD31+ were 1.72 ± 0.88%, 0.89 ± 0.37%, and 0.58 ± 0.39%, respectively. BDCM had significantly higher blood vessel density than FSG. * indicates p<0.05.

Quantification of cells within the wound bed indicates a significant increase of cellular density within the FSG (59.27 ± 2.54%) and BDCM (51.36 ± 9.21%)-treated wounds compared to the ColGAG (29.02 ± 11.05%)-treated wounds (Figure 5A). Additionally, immune cell infiltration was quantified, and the percent area of CD45+ cells was 8.11 ± 6.91%, 6.02 ± 5.22%, and 18.51 ± 16.62% for BDCM-, ColGAG-, and FSG-treated wounds, respectively.

The quantification of collagen indicates a significant difference between percent area collagen in the wound bed between the BDCM- and FSG-treated wounds and the BDCM- and ColGAG-treated wounds. The percent area collagen was 23.18 ± 5.21%, 7.94 ± 7.34%, and 4.27 ± 6.10% for BDCM-, ColGAG-, and FSG-treated wounds, respectively (Figure 5B). Bovine dermal collagen matrix, ColGAG, and FSG percent area CD31+ were 1.72 ± 0.88%, 0.89 ± 0.37%, and 0.66 ± 0.39%, respectively. BDCM had significantly higher blood vessel density than FSG (Figure 5C, p=0.0268).

Dermal matrix persistence and definitive closure

**Figure 6:**
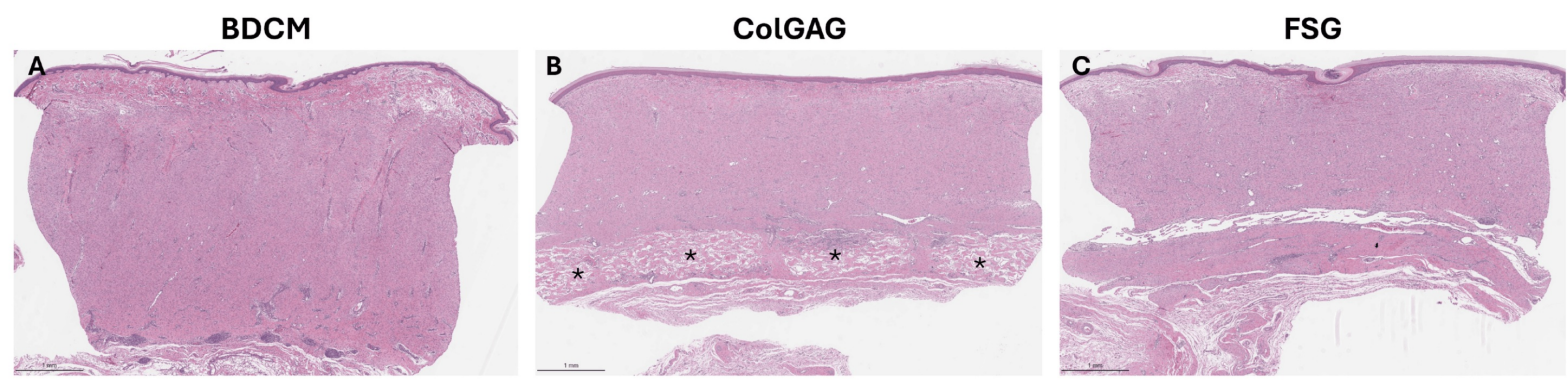
Dermal matrix persistence and definitive closure Hematoxylin & eosin (H&E) was used to evaluate dermal matrix persistence and autograft integration at 35 days post-autograft application. Representative images of H&E-stained tissue sections for integration of the dermal matrices and autograft take for bovine dermal collagen matrix (BDCM) (A), collagen-glycosaminoglycan matrix (ColGAG) (B), and fish skin graft (FSG)-treated wounds (C) indicate the seamless transition between autograft and wound bed. In the ColGAG-treated wounds, the dermal matrix persisted and is seen at the base of the wound bed, indicated by * (B) (scale bar = 1 mm). No detectable fragments of matrix were visualized for BDCM and FSG-treated wounds.

BDCM-treated wounds had good integration of the autograft with the newly generated tissue on Day 42 (Figure 6). Only small remnant BDCM fragments were detected at the end of the study. ColGAG-treated wounds formed a distinct dermal tissue structure that descended from the surface of the wound to the base of the wound near the adipose layer (Figure 6B). No integration of the ColGAG matrix with the autograft was present, as a layer of new tissue existed between the graft and persisting ColGAG material. No fragments of remaining FSG were observed. No breakdown in autografts was observed for areas with graft take observed at seven days post-application for all wounds. 

Wound area and contraction

**Figure 7:**
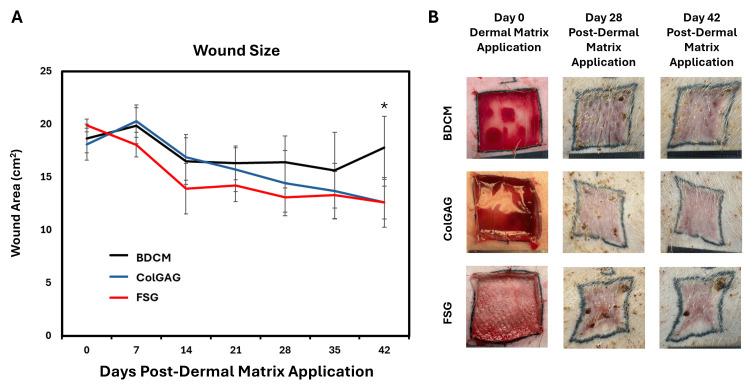
Wound size and appearance The outline of each wound was traced over 42 days for bovine dermal collagen matrix (BDCM) (black line), collagen-glycosaminoglycan matrix (ColGAG) (blue line), and fish skin graft (FSG)-treated wounds (red line), and planimetry was used to determine wound size and contraction. A significant difference in wound size was detected between BDCM and ColGAG-treated wounds and BDCM and FSG-treated wounds at 42 days. No statistical differences were detected at any other time point. Data were reported as mean ± standard deviation; * indicates p<0.05 (A). Representative images for wound appearance at the time of initiation, mid-point, and end of study for BDCM, ColGAG, and FSG-treated wounds highlight findings of planimetry results (B).

The area of the wound was measured at 7, 14, 28, 35, and 42 days post-dermal matrix application (Figure 7). At seven days, the wound areas were 19.88 ± 1.72 cm^2^, 20.31 ± 1.52 cm^2^, and 18.07 ± 1.17 cm^2^, for BDCM-, ColGAG-, and FSG-treated wounds, respectively. Over the course of the study, the wound areas treated with BDCM, ColGAG, and FSG reduced in size to 17.80 ± 2.97 cm^2^, 12.62 ± 1.55 cm^2^, and 12.63 ± 2.34 cm^2^, respectively. At 42 days, on average, BDCM-treated wounds contracted 7.16 ± 14.08%, ColGAG-treated wounds contracted 33.57% ± 11.31%, and FSG-treated wounds contracted 36.59% ± 11.70%. BDCM-treated wounds had a significantly lower percent contraction as compared to FSG-treated wounds (p=0.0073) and ColGAG-treated wounds (p=0.0145).

## Discussion

Dermal matrices are commonly used in patients with full-thickness wounds to generate a vascularized dermal-like base to improve autografting outcomes. Most commonly, dermal matrix application and autografting are performed in separate procedures. The dermal matrix is applied following the excision of necrotic/non-viable tissue, and in a subsequent surgery, the newly generated vascularized tissue is autografted. Generating a vascularized wound bed capable of supporting an autograft directly impacts the time to achieve definitive closure for the patient. This is critical, as a shorter duration for complete wound closure may reduce the risk of complications associated with prolonged healing, including infection and scarring [6-8]. 

In this pre-clinical work, full-thickness wounds treated with BDCM had, on average, a greater percentage of autograft take at 7 days compared to ColGAG- and FSG-treated wounds, 97%, 85%, and 68%, respectively, with low standard deviation and coefficient of variation. These results translated into a greater percentage of re-epithelialization and increased incidence of wound closure at an earlier timeframe. By Day 21, all BDCM-treated wounds were healed, whereas portions of ColGAG and FSG wounds remained open. Additionally, no infection was reported for the BDCM- or FSG-treated wounds.

Over the duration of the study, a difference in wound area was observed, with a significant difference detected on Day 42 for wounds treated with BDCM compared to ColGAG and FSG. This difference may be due to variations in the modulation of the wound bed, the rate of definitive closure, or a combination of both factors.

Histological analysis highlights distinct wound healing responses among the three dermal matrices. During the acute healing phase, BDCM-treated wounds demonstrated significantly greater collagen presence, likely attributed to increased matrix thickness, new collagen synthesis, and material degradation dynamics. This suggests a favorable environment for modulating granulation tissue deposition and contraction. The mean percent area of CD31+ cells correlated with autograft take, emphasizing the critical role of revascularization in graft acceptance and the timeline for definitive closure. While no significant differences were observed in overall inflammatory cell presence, the higher average abundance of CD45+ cells in FSG-treated wounds suggests a more pronounced immune response compared to BDCM and ColGAG-treated wounds.

Histopathological findings for ColGAG and FSG in this study align with previously reported data. Preclinical studies evaluating ColGAG have demonstrated an initial wave of inflammatory cells and cellular infiltration into the lower portion of the collagen matrix by Day 7, with full cellular incorporation and revascularization occurring across the matrix within 21-28 days post-implantation [19,20]. Human biopsy data further support a similar timeline for matrix repopulation [21]. For FSG-treated burn wounds in a porcine model, it was found that the matrix integrated with minimal residual material present, resulting in excessive granulation tissue formation [22].

Outcomes from this pre-clinical study are likely attributable to a combination of biological composition and processing techniques used in material manufacturing. While all three matrices are collagen-based, differences in the age and tissue source of the collagen can impact their regenerative potential and inflammatory profile. The bovine dermal collagen matrix is composed entirely of highly purified and structurally preserved type I and type III collagens derived from the young bovine dermis. This composition provides biological signaling cues that stimulate critical early wound healing processes and angiogenic signals to promote new blood vessel formation [13,23]. Additionally, collagen from younger tissue features a looser fibril structure, which enhances cell infiltration and facilitates tissue remodeling [24,25]. 

In addition to composition, differences in bioengineering processing techniques further distinguish the three matrices, influencing key characteristics such as pore structure, porosity, mechanical strength, and resorption profile. Lyophilization is essential for creating defined pore structures that enable the cells to repopulate the matrix. Crosslinking is vital as it is a tool that can be used to control mechanical properties and degradation rates [12]. Careful selection of crosslinking agents is crucial, as insufficient crosslinking may be ineffective, while excessive crosslinking can inactivate biological materials by denaturing or masking collagen peptide sequences essential for cell binding [26]. Additionally, over-crosslinking may trigger a foreign body response post-implantation [27].

This study has limitations, primarily due to its pre-clinical design. Although the porcine model is commonly used in wound healing research because of the strong similarities between porcine and human skin and the ability to assess within-subject controls, it does not completely replicate the complexity of human conditions. Additionally, the number of wounds evaluated was limited by animal welfare considerations. While a higher mean autograft take was observed for BDCM, statistical differences were not found due to the small sample size and variability in autograft take with ColGAG and FSG. Furthermore, the study was short-term and did not assess long-term effects on tissue formation, remodeling, and contraction outcomes.

## Conclusions

The findings of this study highlight the advantages of bovine dermal collagen matrix (BDCM) in promoting rapid cellular infiltration and vascularization, facilitating early autografting within seven days in full-thickness wounds. Compared to ColGAG and FSG, BDCM demonstrated higher and more consistent autograft take, suggesting its effectiveness as a reliable dermal matrix for early definitive wound closure. Additionally, BDCM-treated wounds exhibited a lower incidence of infection and reduced wound contraction, further supporting its clinical benefits. Given that traditional dermal matrices can extend the wound healing process by 14-28 days, the ability of BDCM to quickly prepare the wound bed for autografting may lead to improved patient outcomes and reduced healthcare costs. These findings position BDCM as a promising advancement in wound care, offering a more efficient and effective approach for deep tissue management. The results from this study warrant clinical evaluation of the material in the management of full-thickness wounds.
