# Correction: Bovine Dermal Collagen Matrix Promotes Vascularized Tissue Generation Supporting Early Definitive Closure in Full-Thickness Wounds: A Pre-clinical Study

**DOI:** 10.7759/cureus.c351

**Published:** 2025-10-07

**Authors:** Katie A Bush, Barbara A Nsiah, Jayson W Jay, Rachel A Penny, Sohail Jahid, Ghaidaa Y Kashgari, Niraj K Doshi, Ian L Valerio

**Affiliations:** 1 Scientific and Medical Affairs, AVITA Medical, Valencia, USA; 2 Research and Development, AVITA Medical, Valencia, USA; 3 Plastic and Reconstructive Surgery, Massachusetts General Hospital, Boston, USA

This article has been corrected to add additional clarification regarding the skin grafting methodology (first and second paragraphs of 'Autografting' section, first paragraph of 'Autograft dosing and take' section).

